# Preoperative physical performance predicts pulmonary complications after coronary artery bypass grafting: a prospective study

**DOI:** 10.1038/s41598-022-15145-2

**Published:** 2022-06-30

**Authors:** Lin Li, Qin Yang, Qi Guo, Dandan Liu, Hui Gao, Yaping Liu

**Affiliations:** 1 Department of Nursing, TEDA International Cardiovascular Hospital, 61, Third Avence, TEDA, Tianjin, 300457 China; 2 Center for Basic Medical Research & Department of Cardiovascular Surgery, TEDA International Cardiovascular Hospital, Chinese Academy of Medical Sciences and Peking Union Medical College, Tianjin, China; 3 Department of Rehabilitation Medicine, Shanghai University of Medicine and Health Sciences, Shanghai, China

**Keywords:** Cardiology, Risk factors, Signs and symptoms

## Abstract

The purpose of this study is to evaluate the relationship between preoperative physical performance (grip strength, gait speed, timed up and go) and postoperative pulmonary complications (PPCs) in patients who have undergone coronary artery bypass grafting (CABG). From September 2019 to August 2021, a total of 497 CABG patients who met the inclusion criteria of this study were examined for grip strength, 4-m gait speed, and timed up and go (TUG) before CABG surgery. Among them, 438 were included in the final analysis. PPCs were classified according to the operational definition of Kroenke et al. and patients with clinically significant PPCs were included in the data analysis. Logistic regression was utilised to analyse the relationship between physical performance and clinically significant PPCs. Besides, the receiver operating characteristic (ROC) curve was applied to analyse the predictive effect of grip strength, gait speed, and TUG on clinically significant PPCs after the CABG procedure. In total, 103 (23.5%) patients developed clinically significant PPCs after CABG. After making adjustments for the European System for Cardiac Operative Risk Evaluation (EuroSCORE) and confounding factors, we established that low grip/weight (*OR* 0.510; 95% *CI* 0.363–0.715), slow gait speed (*OR* 0.619; 95% *CI* 0.517–0.741), and prolonged TUG (*OR* 1.617; 95% *CI* 1.379–1.895) were all independently correlated with clinically significant PPCs after CABG. The ROC curve analysis indicated that the area under the ROC curve of the integrated model of the three indicators (*AUC* 0.792 vs. 0.682, 0.754, 0.765) was larger than that of the model with a single indicator. Besides the predictive effect of the integrated model was superior to the models using grip/weight, gait speed, or TUG alone. Physical performance, including grip/weight, gait speed, and TUG, is a predictive factor for PPCs in CABG patients, and can be used in preoperative evaluations to and help improve the management of high-risk patients.

## Introduction

Postoperative pulmonary complications (PPCs) are the main cause of morbidity and mortality in patients undergoing coronary artery bypass grafting (CABG), which prolong intensive care unit (ICU) stay and the economic burden of the patients^1–3^. Physical decline, a dynamic process that can be prevented early, may occur not only in the elderly but also in people under 65 years old with a variety of diseases^4^. It was reported that 20–50% patients with cardiac diseases showed decline in physical function^5^. At present, the existing preoperative risk assessment, such as European system for cardiac operative risk evaluation (EuroSCORE) and the Society of Thoracic Surgeons (STS) score, can not comprehensively assess the “biological age” and functional ability of patients. Objective physical evaluation has clinical significance in the surgical population, which can optimize the existing risk assessment and help to develop early intervention for high-risk patients to promote postoperative recovery^5,6^. Moreover, the American College of Surgeons and American Geriatrics Society suggests that preoperative evaluation of patients should be improved^7^.

Physical performance can be assessed in many ways, including grip strength measurement that reflects muscle strength, gait speed measurement reflecting mobility, and timed up and go (TUG) test showing dynamic balance^5,8^. Previous studies have reported that preoperative gait speed is independently associated with dyspnea, poor health status and prognosis in patients with pulmonary fibrosis^9^. However, other study showed that gait speed was not associated with pneumonia after liver transplant^10^. Preoperative TUG is a predictor of postoperative complications including respiratory complications and 1-year mortality after cardiac surgery and colorectal surgery^11^, whereas in the elderly patients with elective surgery, the time-consuming TUG was found to be unrelated to any postoperative complications^12^. In patients with congenital heart disease, the predicted value of FEV_1_ increased by 0.74% when the grip strength increased by 1 kg, and the increase in grip strength also enhanced oxygen intake in these patients, which reduced the risk of lung disease^13^. Nevertheless, in the general population, grip strength was found not to be associated with respiratory diseases^14^. In CABG patients, whether different preoperative physical performance can be used to predict the risk of PPCs remains elusive.

Hence, we aimed to explore the correlation between preoperative physical performance (grip strength, gait speed and TUG) and in-hospital PPCs in patients undergoing CABG, and further explore the predictive effect of the above indicators on PPCs.

## Methods

### Study design

The registered name of our trial was Establishment of Perioperative Clinical Rehabilitation System for Cardiac Surgery and the clinical trial number was ChiCTR1800018465. Patients undergoing CABG from September 2019 to August 2021 were enrolled in this prospective cohort study. Preoperative evaluation was performed, with lung function and inspiratory muscle strength test, international physical activity questionnaire short form (IPAQ-SF), activities of daily life (ADL), and EuroSCORE. Preoperative physical performance indicators (grip strength, gait speed and TUG) were analyzed to determine whether they are the influencing factors of in-hospital clinically significant PPCs after CABG.

### Inclusion and exclusion criteria

**Figure 1:**
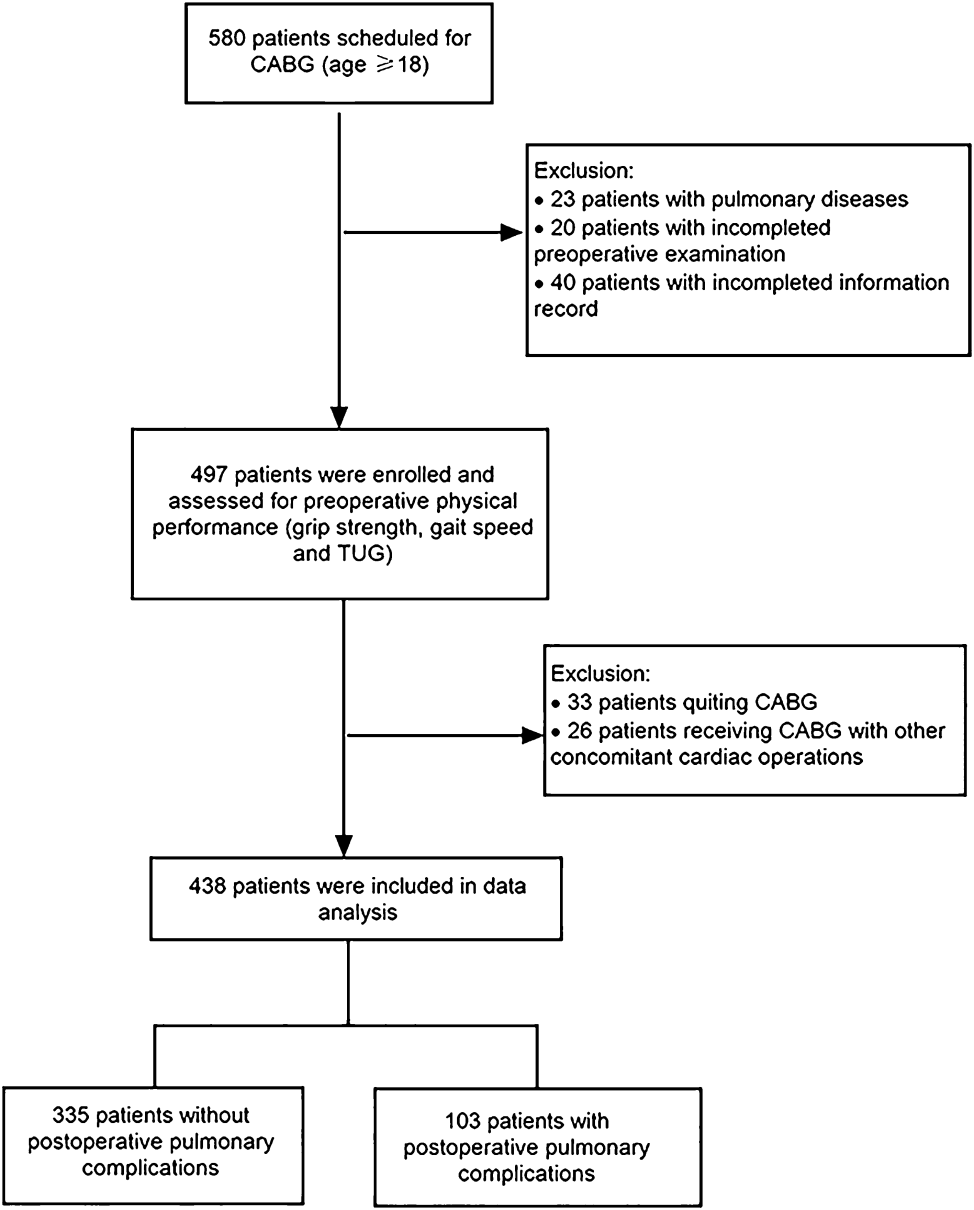
Flow diagram of patient selection and study design.

Patients aged over 18 years old and scheduled for isolated CABG were eligible for inclusion. Patients were excluded if they were (1) unable to complete physical performance assessment, such as those with severe trauma, arthritis or unable to walk independently, Parkinson, amputation, visual impairment, or communication disorders; (2) scheduled for CABG with other concomitant cardiac operations, such as valve surgery or ventricular aneurysm; (3) complicated with pulmonary disease, such as chronic obstructive pulmonary disease (COPD), pneumonia, pulmonary atelectasis, pleural effusion, neoplasms or pneumonectomy; (4) having incomplete data. The flow diagram (Fig. 1) illustrates patient selection and study design.

### Physical performance

To ensure consistency of results, the same team measured the physical performance factors of grip strength, gait speed, and TUG for all patients. Grip strength is an indicator of muscle health, and is widely used in clinical practice^15^. We used a handheld dynamometer (GRIP-D, Takei Ltd., Niigata, Japan) to measure grip strength. Participants were given two opportunities to exert maximum force using their dominant hand, with a 5–10 s rest between attempts. We recorded the mean grip strength value and the grip strength was corrected by the patient’s body weight^14,16^. Gait speed represents mobility and is measured by walking a 4-m distance^17^. Patients were asked to walk twice at a regular speed, and the mean time was recorded. The TUG test, which evaluates balance and lower extremity muscle strength, measures the time taken by patients to stand up from a seated position, walk 3 m, around a marker, and sit back down^18^. Patients are permitted to use a walker during the 4-m walking and TUG tests. Every project was carried out by one trained staff member to complete the data collection of all the subjects. All assessments and tests were completed three days before surgery.

### Outcomes

All operations were performed by the same surgical team, and all surgeons had more than 5 years of experience, thereby avoiding bias. Two blinded independent researchers assessed the occurrence of PPCs in patients with CABG by applying the operational definitions of Kroenke et al. (see Additional File 1: Table S1)^19^. Besides, the researchers evaluated the occurrence of PPCs every day after surgery until discharge. PPCs were regarded as subclinical (e.g., Grade I PPC) when there were only imaging abnormalities but no clinical symptoms or auscultation changes. Clinically significant PPCs were defined as the presence of two or more indications of Grade II PPCs or at least one indication of Grade III or Grade IV PPCs^20^. Disagreements were resolved by consensus or in consultation with a third researcher/senior supervisor. Patients with clinically significant PPCs were included in the analysis.

### Covariates

IPAQ-SF was used to evaluate the frequency of physical activity of different intensity and the cumulative time of each day in the past week, and the metabolic equivalent MET min was calculated to quantify the amount of activity per-week^21^. According to the weekly activity, patients can be categorized into high (> 3000 MET min), medium (600–3000 MET min) and low (< 600 MET min) activity group.

We assessed the degree of preoperative dependence in CABG patients by ADL. Over-all performance in bathing, feeding, toileting, walking, dressing and transferring was recorded^22^. The total score is 100. The higher the score, the better the independence.

EuroSCORE is often used to evaluate the risk of cardiac surgery^23^. The total score was calculated from patient, cardiac, and operation related factors, and divided into low risk (0–2 points), medium risk (3–5 points) and high risk (≥ 6 points).

### Statistical analysis

Continuous normal distribution variables were expressed as *mean* ± *SD* and analyzed with student’s test. The continuous non-normal distribution variables were analyzed by median (25th–75th percentiles) and Mann–Whitney test. The categorical data and grade data were described as absolute number (percentage) and analyzed with chi square test or Mann–Whitney test. Logistic regression was used to analyze the relationship between grip/weight, gait speed, TUG and PPCs. The confounding factors were introduced into the model by forced entry method to evaluate the correlation at different levels. Model 1 was adjusted for age, gender and body mass index (BMI). On the basis of model 1, model 2 was further adjusted for heart rate, smoker, EuroSCORE, IPAQ-SF, ADL, inspiratory muscle strength, MVV, carbamide, creatinine, hypertension, diabetes, hyperlipidemia, operation time, duration of mechanical ventilation, IABP, blood transfusion. The results were expressed as odds ratio (*OR*) and 95% confidence interval (*CI*). We used the receiver operating characteristic (*ROC*) curve to analyze the predictive power of grip/weight, gait speed, and TUG on PPCs, and the predictive power of the combined probability of the three variables on PPCs. The best cutoff value was determined. All data were analyzed by *SPSS v26.0* (SPSS Inc, China). *P* value less than 0.05 was considered as statistically significant.

### Ethics approval and consent to participate

This study was approved by the Clinical Research Ethics Committee at TEDA International Cardiovascular Hospital (2018-0626-4). All participants provided informed consent to participate in the study.

## Results

The mean age of the 438 patients was 63.36 ± 8.39 years. Among the 438 patients, 124 (28.3%) were female and 314 (71.7%) were male, 93.2% of them were in NYHA functional class I–II, 57.1% with medium IPAQ score. The overall mean ADL score was 87.33 ± 7.47, and the overall median EuroSCORE was 4.82 ± 1.85. For male, the median overall grip/weight was 0.45 ± 0.10 kg/kg, gait speed was 0.99 ± 0.19 m/s, and TUG was 8.04 ± 2.54 s. The overall grip/weight, gait speed, and TUG of the female patients were 0.32 ± 0.09 kg/kg, 0.91 ± 0.18 m/s, 9.31 ± 2.50 s, respectively.

PPCs occurred in 40.4% patients, among them 8.0% (35 cases) grade I, 13.5% (59 cases) grade II, 15.1% (66 cases) grade III, and 3.9% grade IV (17 cases). The patients were divided into control group and clinically significant PPCs group (Table 1). In patients with clinically significant PPCs, there were 2.1% expectoration (9 cases), 11.2% hypoxemia (49 cases), 6.4% atelectasis (28 cases), 6.8% hypercarbia (30 cases), 5.5% pleural effusion (24 cases), 3.9% pneumothorax (17 cases), 10.0% suspected or proved pneumonia (44 cases), 3.9% ventilatory failure (17 cases) and 0.9% reintubation (4 cases). Compared with the control group, patients with clinically significant PPCs were older, more often female and smoker, lower in heart rate, carbamide, and creatinine. Patients with clinically significant PPCs also had lower IPAQ and ADL level, higher EuroSCORE, longer surgery and mechanical ventilation time, higher 24 h blood glucose level, and longer hospitalization. The rate of IABP use and postoperative blood transfusion were higher in clinically significant PPCs group. In the clinically significant PPCs group, preoperative MVV and maximum inspiratory muscle were significantly lower than the control group. The overall level of physical performance in PPCs group was worse, evidenced as decreased grip/weight and gait speed as well as increased TUG (*P* < 0.05) (Table 2).

**Table 1:** Clinical characteristics of the patients with and without clinically significant PPCs.

Characteristics	Control (*n* = 335)	Clinically significant PPCs (*n* = 103)	Statistics	*P*
**General characteristic**				
Age (y)	62.84 ± 8.39	65.41 ± 8.10	*t* = − 2.340	0.020
Female (n, %)	85 (25.4)	39 (37.9)	*X*^2^ = 6.056	0.014
BMI (kg/m^2^)	25.84 ± 3.29	25.38 ± 3.18	*t* = 1.270	0.205
Heart rate (bpm)	73.55 ± 11.66	69.77 ± 11.64	*t* = 2.883	0.004
Farmer (n, %)	119 (35.5)	49 (47.6)	*X*^2^ = 6.631	0.085
Illiteracy (n, %)	27 (8.1)	6 (5.8)	*Z* = − 1.719	0.143
Smoker (n, %)	103 (30.7)	48 (46.6)	*X*^2^ = 8.767	0.003
Drinker (n, %)	82 (24.5)	22 (21.4)	*X*^2^ = 0.423	0.515
EuroSCORE	4.62 ± 1.86	5.46 ± 1.69	*t* = − 4.094	< 0.001
**Laboratory examination**				
Hemoglobin (g/l)	135.62 ± 14.93	134.32 ± 14.55	*t* = 0.779	0.436
Serum Albumin (g/dl)	4.23 ± 0.50	4.14 ± 0.45	*t* = 1.611	0.108
Carbamide (mmol/l)	5.93 ± 1.93	5.51 ± 1.56	*t* = 2.018	0.044
Creatinine (umol/l)	72.28 ± 23.08	66.90 ± 16.65	*t* = 2.196	0.029
**Cardiorespiratory function**				
Angina pectoris (n, %)				
Stable angina pectoris	22 (6.6)	5 (4.9)		
Unstable angina pectoris	278 (83.0)	88 (85.4)	*X*^2^ = 2.135	0.545
ST-elevation MI	15 (4.5)	2 (1.9)		
Non ST-elevation MI	20 (6.0)	8 (7.8)		
LVEDD (mm)	47.91 ± 4.48	47.74 ± 5.08	*t* = 0.337	0.736
LADs (mm)	37.41 ± 3.82	37.29 ± 4.03	*t* = 0.270	0.788
Mitral E/A	0.86 ± 0.35	0.88 ± 0.42	*t* = − 0.401	0.689
LVEF	60.72 ± 8.65	60.61 ± 9.22	*t* = 0.109	0.913
NYHA (n, %)				
I	44 (13.1)	12 (11.7)		
II	262 (80.3)	83 (80.6)	*Z* = − 0.539	0.590
III	22 (6.6)	8 (7.8)		
Lung function (%predicted)				
VC	79.39 ± 18.44	78.29 ± 20.41	*t* = 0.514	0.607
FVC	84.59 ± 18.17	82.84 ± 20.94	*t* = 0.828	0.408
FEV_1_	85.83 ± 17.71	82.69 ± 20.35	*t* = 1.520	0.129
FEV_1_/FVC	107.07 ± 12.60	105.50 ± 13.93	*t* = 1.076	0.282
MVV	76.04 ± 24.89	68.30 ± 20.75	*t* = 2.865	0.004
Inspiratory muscle strength				
Pi-max (cmH_2_O)	88.61 ± 26.05	80.69 ± 25.13	*t* = 2.721	0.007
**Comorbidity**				
Hypertension (n, %)	250 (74.6)	71 (68.9)	*X*^2^ = 1.305	0.253
Diabetes (n, %)	160 (47.8)	42 (40.8)	*X*^2^ = 1.547	0.214
Hyperlipidemia (n, %)	101 (30.1)	32 (31.1)	*X*^2^ = 0.031	0.859
Valvular disease (n, %)	78 (23.3)	25 (24.3)	*X*^2^ = 0.043	0.836
Cerebrovascular disease (n, %)	102 (30.4)	36 (35.0)	*X*^2^ = 0.740	0.390
**Surgical procedure data**				
Extracorporeal circulation (n, %)	268 (80.0)	88 (85.4)	*X*^2^ = 1.530	0.216
Operation time (min)	231.43 ± 59.47	264.89 ± 81.58	*t*′ = − 3.860	< 0.001
Number of grafts	3.60 ± 0.85	3.70 ± 0.84	*t* = − 1.005	0.316
IABP (n, %)	4 (1.2)	9 (8.7)	*X*^2^ = 13.058	< 0.001
Duration of mechanical ventilation (h)	9.47 ± 6.94	35.55 ± 74.42	*t*′ = − 3.552	0.001
24 h Blood glucose level (mmol/l)	9.04 ± 2.10	9.93 ± 3.03	*t*′ = − 2.767	0.006
Blood transfusion (n, %)	121 (36.1)	49 (47.6)	*X*^2^ = 4.352	0.037
Lengh of stay (day)	8.73 ± 3.39	12.74 ± 9.12	*t*′ = − 4.369	< 0.001

*BMI* body mass index, *MI* myocardial infarction, *LVEDD* left ventricular end diastolic diameter, *LADs* left atrial diameter, *Mitral E/A* mitral peak velocity of early filling (E) to mitral peak velocity of late filling (A), *LVEF* left ventricular ejection fraction, *NYHA* New York Heart Association, *EuroSCORE* European system for cardiac operative risk evaluation, *VC* inspiratory vital capacity, *FVC* forced vital capacity, *FEV*_*1*_ forced expiratory volume in the first second of expiration, *MVV* maximal voluntary ventilation, *IABP* intra-aortic balloon pump.

**Table 2:** Physical performance of the patients with and without clinically significant PPCs.

Characteristics	Control (*n* = 335)	Clinically significant PPCs (*n* = 103)	Statistics	*P*
**Measurement**				
Grip/weight (kg/kg)	0.43 ± 0.11	0.36 ± 0.11	*t* = 5.603	< 0.001
Gait speed (m/s)	1.00 ± 0.17	0.85 ± 0.21	*t* = 7.707	< 0.001
TUG (s)	7.79 ± 1.77	10.40 ± 3.65	*t*′ = − 7.014	< 0.001
**Questionnaire**				
IPAQ high level (n, %)	72 (21.5)	14 (13.6)	*Z* = − 2.278	0.023
ADL	87.78 ± 6.96	85.87 ± 8.81	*t* = 2.272	0.024

*TUG* timed up and go, *IPAQ* international physical activity questionnaire, *ADL* activities of daily life.

In the univariate logistic regression models, grip/weight and gait speed were negatively correlated with PPCs, while TUG was positively correlated with clinically significant PPCs (Table 3). In the multivariate logistic regression models, the OR of grip/weight, gait speed and TUG changed slightly whereas their correlation with clinically significant PPCs in CABG patients was still statistically significant (Table 4).

**Table 3:** Univariate logistic regression models of influencing factors and clinically significant PPCs.

Variables	*B*	Standard error	Wald *X*^*2*^	*P*	OR (95% CI)
Gait/weight (kg/kg × 10)	− 0.595	0.113	27.498	< 0.001	0.552 (0.442–0.689)
Grip speed (m/s × 10)	− 0.520	0.077	46.033	< 0.001	0.594 (0.511–0.691)
TUG (s)	0.455	0.060	57.805	< 0.001	1.576 (1.402–1.773)
Age (y)	0.033	0.014	5.359	0.021	1.034 (1.005–1.063)
Female	0.583	0.239	5.970	0.015	1.792 (1.122–2.862)
BMI (kg/m^2^)	− 0.045	0.035	1.607	0.205	0.956 (0.892–1.025)
Heart rate (bpm)	− 0.029	0.010	8.032	0.005	0.971 (0.952–0.991)
Smoker	0.676	0.230	8.613	0.003	1.966 (1.252–3.087)
EuroSCORE	0.244	0.062	15.569	< 0.001	1.276 (1.130–1.440)
**IPAQ**				0.780	
Low	1.00 (reference)				
Medium	− 0.368	0.262	1.972	0.160	0.692 (0.414–1.157)
High	− 0.809	0.363	4.970	0.026	0.445 (0.219–0.907)
ADL	− 0.031	0.014	4.945	0.026	0.969 (0.943–0.996)
Inspiratory muscle strength (cmH_2_O)	− 0.012	0.005	7.189	0.007	0.988 (0.979–0.997)
MVV (%predicted)	− 0.014	0.005	7.943	0.005	0.986 (0.976–0.996)
Carbamide (mmol/l)	− 0.148	0.073	4.060	0.044	0.863 (0.747–0.996)
Creatinine (umol/l)	− 0.017	0.007	5.255	0.022	0.984 (0.970–0.998)
Hypertension	− 0.282	0.247	1.300	0.254	0.754 (0.465–1.225)
Diabetes	− 0.284	0.228	1.542	0.214	0.753 (0.481–1.178)
Hyperlipidemia	0.043	0.244	0.031	0.859	1.044 (0.647–1.684)
Operation time (min)	0.007	0.002	18.084	< 0.001	1.007 (1.004–1.010)
Duration of mechanical ventilation (h)	0.062	0.012	27.956	< 0.001	1.064 (1.040–1.089)
IABP	2.070	0.612	11.431	0.001	7.923 (2.387–26.301)
Blood transfusion	0.473	0.228	4.314	0.038	1.605 (1.027–2.508)

*TUG* timed up and go, *BMI* body mass index, *IPAQ* International physical activity questionnaire, *ADL* activities of daily life, *MVV* maximal voluntary ventilation, *IABP* intra-aortic balloon pump.

**Table 4:** Multivariate logistic regression models of physical performance and clinically significant PPCs.

Variables	Model 1^a^	*P*	Model 2^b^	*P*
	*OR* (95% *CI*)		*OR* (95% *CI*)	
Grip/weight (kg/kg)	0.471 (0.353–0.630)	< 0.001	0.510 (0.363–0.715)	< 0.001
Gait speed (m/s)	0.591 (0.503–0.693)	< 0.001	0.619(0.517–0.741)	< 0.001
TUG (s)	1.641 (1.436–1.876)	< 0.001	1.617 (1.379–1.895)	< 0.001

*TUG* timed up and go.

^a^Model 1, age, gender, BMI.

^b^Model 2, age, gender, BMI, Heart rate, Smoker, EuroSCORE, IPAQ, ADL, Inspiratory muscle strength, MVV, Carbamide, Creatinine, Hypertension, Diabetes, Hyperlipidemia, Operation time, Duration of mechanical ventilation, IABP, Blood transfusion.

**Figure 2:**
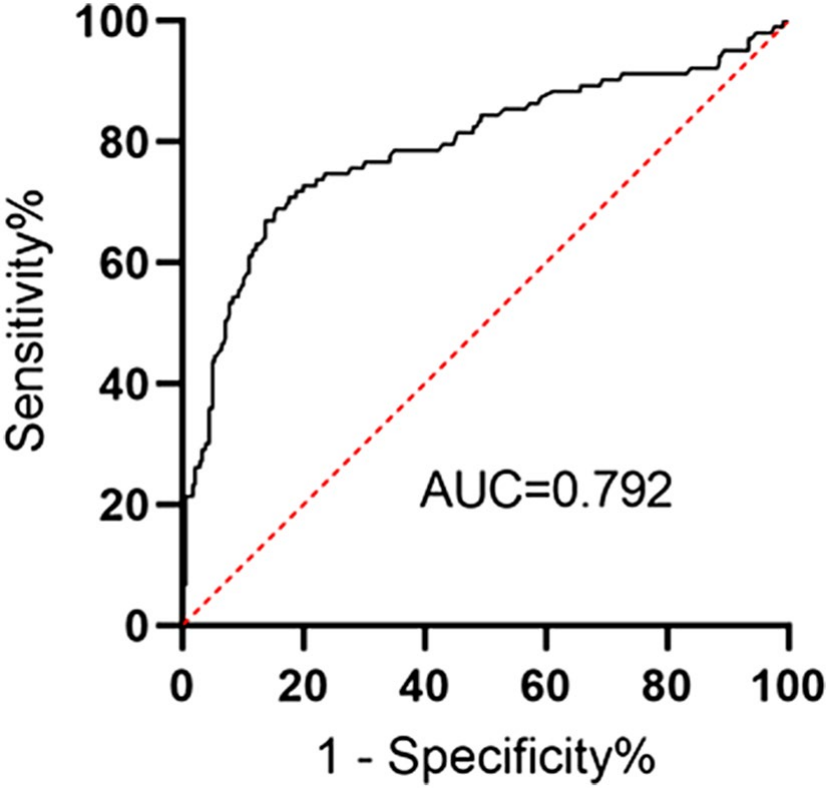
Integrated sensitivity and specificity of grip/weight, gait speed and TUG for PPCs prediction in CABG patients.

Using the *ROC* curve, we found that the area under *ROC* curve (*AUC*) of grip/weight was 0.682, and the sensitivity and specificity were low. The AUC area of gait speed and TUG was more than 0.75. Compared with gait speed, the sensitivity (66.0% vs 64.1%) and specificity (83.3% vs 82.4%) of TUG were higher. The predictive value of the integrated model that involve three indicators was the best (AUC, 0.792 vs. 0.682, 0.754, 0.765), compared with the model involving grip/weight, gait speed or TUG alone. The cutoff value was 0.285, and the integrated model had 68.9% sensitivity and 84.8% specificity in predicting clinically significant PPCs in CABG patients (Table 5, Fig. 2).

**Table 5:** AUC and cutoff value of physical performance indicators for predicting clinically significant PPCs in CABG patients.

	AUC (95%*CI*)	*P*	Cutoff	Sensitivity (%)	Specificity (%)
Grip/weight (kg/kg)	0.682 (0.621–0.743)	< 0.001	0.365	59.2	72.5
Gait speed (m/s)	0.754 (0.693–0.816)	< 0.001	0.845	64.1	82.4
TUG (s)	0.765 (0.707–0.823)	< 0.001	9.110	66.0	83.3
Integrated model	0.792 (0.734–0.849)	< 0.001	0.285	68.9	84.8

*TUG* timed up-and-go, *AUC* the are under the receiver operating characteristic curve.

## Discussion

In this study, we explored the correlation between preoperative physical performance and PPCs in CABG patients. Our results showed that grip/weight, gait speed, and TUG were all independently associated with in-hospital PPCs after CABG and could serve as risk predictors. Previous studies regarding the predictive value of preoperative physical activity for postoperative complications in cardiac surgery patients yielded inconsistent conclusions. For example, in patients receiving transcatheter aortic valve replacement, preoperative physical decline, as indicated by grip strength, gait speed, and Katz ADL survey score, was found to be unable to predict postoperative acute kidney injury, stroke, and vascular complications^24^. Other studies using gait speed or TUG as the physical activity indicator suggested that gait speed and TUG have good predictive power for prolonged mechanical ventilation, infection, and reoperation in patients underwent cardiac surgery^11,25^. In addition to the difference in study population, the inconsistence may also be attributed to the differences in evaluation methods. Indeed, at present, there are no unified methods for physical performance measurement. In this study, we jointly used grip strength, gait speed, and TUG as the indicators of physical performance, which in comparison with using only one or two of them more comprehensively reflects the physical function of the patients.

There are many factors influencing the incidence of PPCs after CABG. It was reported that patients with COPD and poor lung function before CABG are more likely to have pneumonia, reintubation, and prolonged ventilation (*P* < 0.001), and FEV_1_ less than 60% of the predicted value is a good predictor of poor prognosis and mortality after CABG^26^. In our study, the patients with or without clinically significant PPCs showed no differences in VC, FVC, FEV_1_, and FEV_1_/FVC. Although, MVV was lower in patients with clinically significant PPCs in univariate analysis, its effect on the incidence of clinically significant PPCs was insignificant after adjusting for multiple factors (*P* > 0.05). A previous study showed that preoperative maximal inspiratory muscle strength, FEV_1_ predicted, FVC predicted, MVV predicted increase after 5-day of preoperative inspiratory muscle training, which significantly reduce the incidence of PPCs after CABG and/or valve surgery^20^. Therefore, preoperative inspiratory muscle strength also has a great influence on PPCs in patients with CABG. Furthermore, EuroSCORE is widely used in risk assessment before cardiac surgery, but it is easy to be affected by age and overestimates the risk of surgery. In addition, EuroSCORE II includes physical performance related indicators such as poor mobility, but the definition of poor mobility is subjective, not objective enough to assess the frailty^27^. Our study showed that EuroSCORE had a weaker prediction effect (AUC, 0.646 vs. 0.792) on clinically significant PPCs than physical performance.

### Relationship between grip strength and PPCs

It is known that grip strength is negatively correlated with all-cause mortality of cardiovascular disease^14^. Choi’s study reported that low grip strength (*OR* 1.15; 95% *CI* 1.08–1.22) could independently predict pneumonia and pulmonary thromboembolism after hip fracture surgery^28^. In patients with esophageal cancer, low grip strength was an independent predictor of postoperative complications (*OR* 4.89; 95% *CI* 1.53–15.67), and the incidence of pneumonia was significantly higher than normal group (*P* = 0.001)^29^. Consistently, our study confirmed that in patients undergoing CABG, poor grip strength (*OR* 0.510; 95% *CI* 0.363–0.715) is an independent risk factor for PPCs. However, Leong et al. found that there was no correlation between grip strength and incidence of pneumonia or COPD^14^. The divergence in results may be due to the differences in population (general vs. CABG), age (50 vs. 63), and physical activity level (high IPAQ score, 46.0% vs. 19.6%). Notably, it was reported that moderate intensity grip strength training could produce antihypertensive effect, promote the balance of autonomic nervous system, attenuate endothelial dysfunction and inflammatory reaction to reduce the risk of adverse events^30^. Whether grip strength training can improve the preoperative lung function to improve the prognosis of CABG patients warrants further studies.

### Relationship between gait speed and PPCs

Gait speed is an effective index to measure frailty, as it reflects muscle function of the lower limbs and represents cardiopulmonary health to a certain extent. A previous study showed that after adjusting the Society of Thoracic Surgeons Predicted Risk of Mortality (STS-PROM) value, the gait speed increased by 0.1 m/s, relative mortality within 30 days after CABG decreased by 14%, and the number of slow walkers who required prolonged mechanical ventilation (> 24 h) was more than double that of the fast walkers^25^. In patients with liver transplantation, Salim and colleagues reported that after adjusting for age and gender, slow gait speed before surgery is negatively associated with aspiration (*OR* 0.12; 95% *CI* 0.02–0.87, *P* = 0.035), and days of intubation (*OR* − 1.14; 95% *CI* − 2.13 to − 0.15, *P* = 0.025), but not associated with postoperative pneumonia (*OR* 0.19; 95% *CI* 0.89–1.08, *P* = 0.077)^10^. Our study established that preoperative slow gait speed (*OR* 0.619; 95% *CI* 0.517–0.741) could predict the incidence of clinically significant PPCs in CABG patients. Since slow gait speed is a manifestation of decreased physiological reserves, muscle atrophy, and declined vasoconstriction and vasodilation function, the bodies of patients with slow gait speed do not provide enough compensation for stress events such as surgery and intubation^10,31^. Damage to airway protection mechanisms and swallowing function as well as the reduction of tissue oxygenation due to neuromuscular imbalance may result in a higher incidence of PPCs. Nevertheless, in a study of 166 patients underwent cardiac surgery, slow gait speed was found to be unrelated to the incidence of postoperative complications (*P* = 0.332)^32^. We believe that gait speed alone can not accurately reflect physical performance, which is related to cachexia, muscle loss and lower BMI. Therefore, more variables should be evaluated to correctly define and identify frailty, thereby providing reliable information for clinicians.

### Relationship between TUG and PPCs

The TUG test is an important indicator of functional status. It is coordinated by the nervous system (sensory input, central processing, and motor control) and the musculoskeletal system (muscles and joints). Compared to patients with TUG ≤ 10 s, cardiac surgery patients with TUG > 10 s exhibited a higher incidence of postoperative respiratory complications. Besides, the predictive effect of TUG was better than that of the Veterans Affairs mortality risk calculator (*AUC* 0.684 vs. 0.552)^11^, which had lower discriminative power than our study. This may be due to the differences in sample size (174 vs. 438), outcome indicators (unspecified postoperative complications vs. PPCs), and BMI (29.0 vs. 25.7). Additionally, another study showed that 58% of the patients enrolled in stage II cardiac rehabilitation exhibit balance abnormality, which increases the incidence of postoperative adverse events^33^. Consistent with these reports, we confirmed that TUG (*OR* 1.617; 95% *CI* 1.379–1.895) is an independent risk factor for clinically significant PPCs in patients with CABG. Nevertheless, Min and colleagues found that TUG > 20 s is not associated with postoperative complications (*P* = 0.084)^12^, which may be related to the lack of a uniform cutoff value for the TUG test. Min et al. defined the cutoff value of TUG as 20 s, while the cutoff value in this study was 9.11 s. Thus, it is very likely that different TUG cutoff values lead to inconsistent results. Other studies asserted that slow TUG, accompanied by hypoxemia, dyspnoea, decreased inspiratory muscle strength, and impaired lung function lead to elevated levels of high-sensitivity C-reactive protein and fibrinogen, thereby promoting systemic inflammatory response^34,35^. This may be the mechanism by which slow TUG increases the risk of PPCs after CABG. Moreover, it is worth mentioning that in an intervention study of CABG patients, two weeks of preoperative rehabilitation was found to effectively improve preoperative functional ability (TUG, six-minute walk distance), avoid postoperative complications, reduce mortality, and improve patient quality of life^36^.

### Cutoff values of efficient physical indicators

The current study also compared the accuracy of single and integrated models of grip/weight, 4-m gait speed, and TUG in predicting the occurrence of clinically significant PPCs in CABG patients. Preoperative grip/weight had poor prediction power for PPCs with 59.2% sensitivity, which might produce 40.8% false negative prediction. Therefore, the optimal cutoff value should be used carefully. In a previous study of CABG patients, compared with grip/weight, postoperative grip recovery was found to be more predictive for the occurrence of complications within 30-day of discharge after CABG^37^. We found that TUG is a better independent predictor of clinically significant PPCs compared with grip/weight and gait speed (*AUC* 0.765 vs. 0.682 and 0.754), with more than 65% sensitivity and specificity. However, it should be noted that only TUG can not accurately reflect preoperative frailty, which needs to be combined with grip strength, gait speed, unintended weight loss, or exhaustion^38^. Our results showed that the AUC area of the integrated model that involve all the three indicators is larger. The prediction sensitivity and specificity of the integrated model was higher than that of grip/weight, gait speed, or TUG alone in predicting clinically significant PPCs. CABG patients who may develop clinically significant PPCs are more accurately identified when the optimal cutoff value of the integrated model is ≥ 0.285. Moreover, previous studies have shown that the predictive effect of twelve independent factors (such as gender, diabetes, and preoperative creatinine) on respiratory failure after cardiac surgery is 0.751, the EuroSCORE is 0.710^39^, and the STS score is 0.670. Nevertheless, adding either the lung function test or COPD classifications does not improve the discrimination of the STS score^40^. Furthermore, in patients undergoing robot-assisted laparoscopic prostatectomy, the AUC of the preoperative diaphragm thickening fraction for predicting PPCs is 0.714^41^. Although the AUC value of 0.792 in our study reflects satisfactory discrimination, values greater than 0.8 are necessary for good discrimination. However, the AUC value we obtained is still higher than the predictive effect of other PPCs indicators. Thus, it has a certain guiding significance for clinical practice. Therefore, adding of grip strength, gait speed and TUG tests, which are easy to conduct, to the existing preoperative evaluation system may further help predicting the risk of postoperative complications and provide more information for the development of targeted preoperative rehabilitation to improve the prognosis of patients who receive CABG.

### Limitations

To our knowledge, this is the first study that evaluates a combination of grip strength, gait speed, and TUG to explore the predictive effect of preoperative physical activity on PPCs in CABG patients. However, there are some limitations to this study. Firstly, we did not measure the preoperative muscle mass (an important indicator of sarcopenia) of the patients. Therefore, it was not clear whether a combination of muscle mass with grip/weight, gait speed, and TUG could more effectively predict PPCs. Besides, we excluded patients with known pulmonary disease, which may lead to high PPCs risk. In future research, we will increase the sample size of such patients and include this factor. Additionally, this is a single centre study with a relatively small number of participants, and it includes only in-hospital PPCs. Further multi-centre studies with more patients are required and prolonged the follow-up times are needed to make the results more broadly representative. The predictive power of the integrated model may be further improve by addressing the above-mentioned limitations.

## Conclusions

Low grip/weight, slow gait speed, and prolonged TUG are associated with increased risk of PPCs after CABG. Use of these physical performance indicators in combination may increase the accuracy of preoperative risk prediction and help improve the management of high-risk patients.

## Supplementary Information

Supplementary Table S1.

## Data Availability

The datasets used and/or analysed during the current study are available from the corresponding author on reasonable request.

## Abbreviations

CABG: Coronary artery bypass grafting
PPCs: Postoperative pulmonary complications
TUG: Timed up and go
ROC: Receiver operating characteristic
ICU: Intensive care unit
EuroSCORE: European system for cardiac operative risk evaluation
IPAQ: International physical activity questionnaire
ADL: Activities of daily life
COPD: Chronic obstructive pulmonary disease
MVV: Maximal voluntary ventilation
OR: Odds ratio
CI: Confidence interval
BMI: Body mass index
MI: Myocardial infarction
LVEDD: Left ventricular end diastolic diameter
LADs: Left atrial diameter
Mitral E/A: Mitral peak velocity of early filling (E) to mitral peak velocity of late filling (A)
LVEF: Left ventricular ejection fraction
NYHA: New York Heart Association
VC: Inspiratory vital capacity
FVC: Forced vital capacity
FEV_1_: Forced expiratory volume in the first second of expiration
IABP: Intra-aortic balloon pump
AUC: Area under the curve
