# Supplementary material for: Preoperative physical performance predicts pulmonary complications after coronary artery bypass grafting: a prospective study
Source: Sci Rep. 2022 Jun 30;12:11103. doi: 10.1038/s41598-022-15145-2 (PMC9246884; doi:10.1038/s41598-022-15145-2)
Supplement: Supplementary file 1 — Supplementary Table S1. [file 41598_2022_15145_MOESM1_ESM.docx]

Additional Table S1. The definition and classification of postoperative pulmonary complications^a^

| **Grade I** |
| --- |
| - Cough, dry |
| - Microatelectasis: abnormal lung findings and temperature >37.5℃ without other documented cause; results of chest radiograph either normal or unavailable |
| - Dyspnea, not due to other documented cause |
| **Gade II** |
| - Cough, productive, not due to other documented cause |
| - Bronchospasm: new wheezing or preexistent wheezing resulting in change therapy |
| - Hypoxemia: alveolar-arterial gradient >29 and symptoms of dyspnea or wheezing |
| - Atelectasis: radiological confirmation plus either temperature >37.5℃ or abormal lung findings |
| - Hypercarbia, transient, requiring treatment, such as naloxone or increased manual or mechanical ventilation |
| - Adverse reaction to pulmonary medication |
| **Grade III** |
| - Pleural effusion, resulting in thoracentesis |
| - Pneumonia, suspected：radiological evidence without bacteriological confirmation |
| - Pneumonia, proved: radiological evidence and documentation of pathological organism by Gram stain or culture |
| - Pneumothorax |
| - Reintubation postoperative or intubation, period of ventilator dependence dose not exceed 48 hours |
| **Grade IV** |
| - Ventilatory failure: postoperative ventilator dependence exceeding 48 hours, or reintubation with subsequent period of ventilator dependence exceeding 48 hours |

^a^Soure: Kroenke *et al*.^[19]^
